# Sexual violence in dating relationships among high school adolescents: a school-based study^*^

**DOI:** 10.1590/1518-8345.8316.4918

**Published:** 2026-10-09

**Authors:** Isaura Barros Alves Pinto, Rita Mafalda Costa Francisco, Franciele Marabotti Costa Leite

**Affiliations:** 1 Universidade Federal do Espírito Santo, Departamento de Enfermagem, Vitória, ES, Brazil.; 2 Instituto de Medicina Social, Universidade do Estado do Rio de Janeiro, Rio de Janeiro, RJ, Brazil.; 3 Departamento do Programa Nacional de Imunizações, Ministério da Saúde, Brasília, DF, Brazil.; 4 Universidade Católica Portuguesa, Faculdade de Ciências Humanas, Lisboa, Portugal.

**Keywords:** Violence, Intimate Partner Violence, Adolescent Health, Epidemiology, Public Health, Nursing.

## Abstract

**Objective:**

to analyze the prevalence of sexual violence in dating relationships among high school adolescents and its association with socioeconomic and behavioral characteristics and experiences of violence.

**Method:**

a cross-sectional, analytical study was conducted among 1,238 adolescents aged 14 to 19 years enrolled in public and private schools who were in romantic relationships at the time of the study. Sampling was by clusters and stratification.

**Results:**

of the 1,238 adolescents in romantic relationships, 7.8% (95% CI: 6.4-9.4) reported having experienced sexual violence in dating. The prevalence was 9.3% among girls and 5.1% among boys. There was a higher likelihood of sexual violence in dating relationships among non-heterosexual adolescents, those who had ever had sexual intercourse, those who used drugs, and those who had experienced bullying and cyberbullying (p<0.05).

**Conclusion:**

it is essential that these findings contribute to the strengthening of educational campaigns in schools, addressing not only abusive behaviors but also the importance of consent, mutual respect, and the construction of secure emotional bonds among adolescents.

## Highlights

**(1)** Prevalence of 7.8% of sexual violence in adolescent dating relationships.

**(2)** Findings reinforce the urgency of addressing violence in youth relationships.

**(3)** Sexual victimization affects adolescents in their first romantic relationships.

**(4)** Sexual violence in dating is associated with drugs, bullying, cyberbullying, ever having sexual intercourse, and non-heterosexuals.

**(5)** Partnerships with health, school, and family promote listening and abuse prevention.

## Introduction

Adolescence marks a period of transition in which interpersonal relationships, especially friendships and romantic relationships, take center stage, while family ties are reconfigured^1^. At this stage, the interaction of individual and social factors can favor the internalization of norms that normalize sexual violence^2^ ^-^ ^3^.

Dating violence (DV), understood as any form of physical, emotional, or sexual aggression by an intimate partner^4^, often manifests itself as a mechanism of control and domination. Among its expressions, sexual violence stands out for involving coercion, harassment, or the imposition of non-consensual sexual acts, constituting a serious violation of human rights^5^. Studies indicate that this violence can occur in non-marital relationships, regardless of sexual orientation^6^.

Epidemiological data illustrate the magnitude of the problem: a survey of Brazilian adolescents^7^ found that 38% reported experiences of sexual violence in dating relationships, including pressure to engage in intimate activities and non-consensual sharing of images. These experiences are correlated with consequences such as depression, post-traumatic stress, and substance use^8^. Furthermore, the normalization of these aggressions, often rooted in gender stereotypes, makes identification and reporting difficult^3^.

Given this scenario, this study aims to analyze the prevalence of sexual violence in dating among high school adolescents and its association with socioeconomic and behavioral characteristics and experiences of violence.

## Method

### Study design

This is a cross-sectional, analytical study conducted in public and private schools in municipalities within the Greater Vitória Metropolitan Area (RMGV) in the state of Espírito Santo.

### Location

The Greater Vitória Metropolitan Area (RMGV, in Portuguese) comprises the municipalities of Cariacica, Fundão, Guarapari, Serra, Viana, Vila Velha, and Vitória. This region is home to approximately 1,880,828 inhabitants, spread over an area of 2,331 km²^9^.

### Period

Data collection took place between March and December 2023.

### Population

The target population of the study was high school adolescents aged 14 to 19 years, regularly enrolled in public or private schools in the RMGV who participated after their guardians accepted the Free and Informed Consent Form (FICF) and the adolescents signed the Informed Assent Form (IAF), after being informed about the objectives of the research and its possible risks and benefits.

### Sample definition

The sampling used a 95% confidence level and a maximum error of 5%. Across strata of municipalities, the maximum error was 10%, with a minimum sample size of 4,416 students. For this study, only adolescents who were in some type of relationship at the time of the interview were selected, totaling a sample of 1,238 adolescents.

Before the data collection stage, the selected schools were invited by email to participate in a seminar presenting the research and subsequently contacted by email/telephone to schedule data collection. It is important to note that, preceding the fieldwork, the team was recruited and trained, and a pilot test was conducted in February 2023. Data collection took place from March to December 2023, using tablets, with adolescents self-responding to a questionnaire in the REDCap (Research Electronic Data Capture) system.

### Study variables

The outcome in the present study was sexual violence in dating relationships, which was dichotomized (yes/no). The variable-sexual violence in dating relationships-was considered affirmative if the adolescent had experienced at least one of the following situations: “your partner caressed you without your consent and against your will, forced you to have sex when you did not want to, prevented you from using condoms, induced you to have an abortion, or any other issue that infringed on your sexual and reproductive rights”.

As for the independent variables, the following sociodemographic variables were analyzed: gender (female/male); age group (≤15 years/≥16 years); cisgender (yes/no); heterosexual (yes/no); race/color (white/black/brown); economic classification measured by the Economic Classification Criteria of the Brazilian Association of Research Companies (ABEP), which classifies into class A/B/C/D/E; has work/employment (no/yes); and religion (none/Catholic/Evangelical/others). The following behavioral variables were also analyzed: sexual intercourse at some point in life (no/yes); current cigarette use (no/yes); current drug use (no/yes) and current alcohol use (no/yes), as well as variables related to experiences of violence: ever experienced cyberbullying (no/yes); ever engaged in cyberbullying (no/yes); ever experienced bullying (no/yes); ever engaged in bullying (no/yes).

### Data processing and analysis

For descriptive statistical analyses, absolute and relative frequencies were calculated. In the bivariate analysis, Pearson’s chi-square test (χ2) was used as assumed, with respective 95% confidence intervals. For crude multivariate analysis and odds ratio adjustment using logistic regression to assess associations between independent variables and dating violence, descriptive and bivariate analysis were initially performed with calculation of the crude odds ratio (OR). Variables with p ≤0.20 were included in the multivariate model adjusted for confounding factors and retained at p ≤ 0.05 (Figure 1).

**Figure 1:**
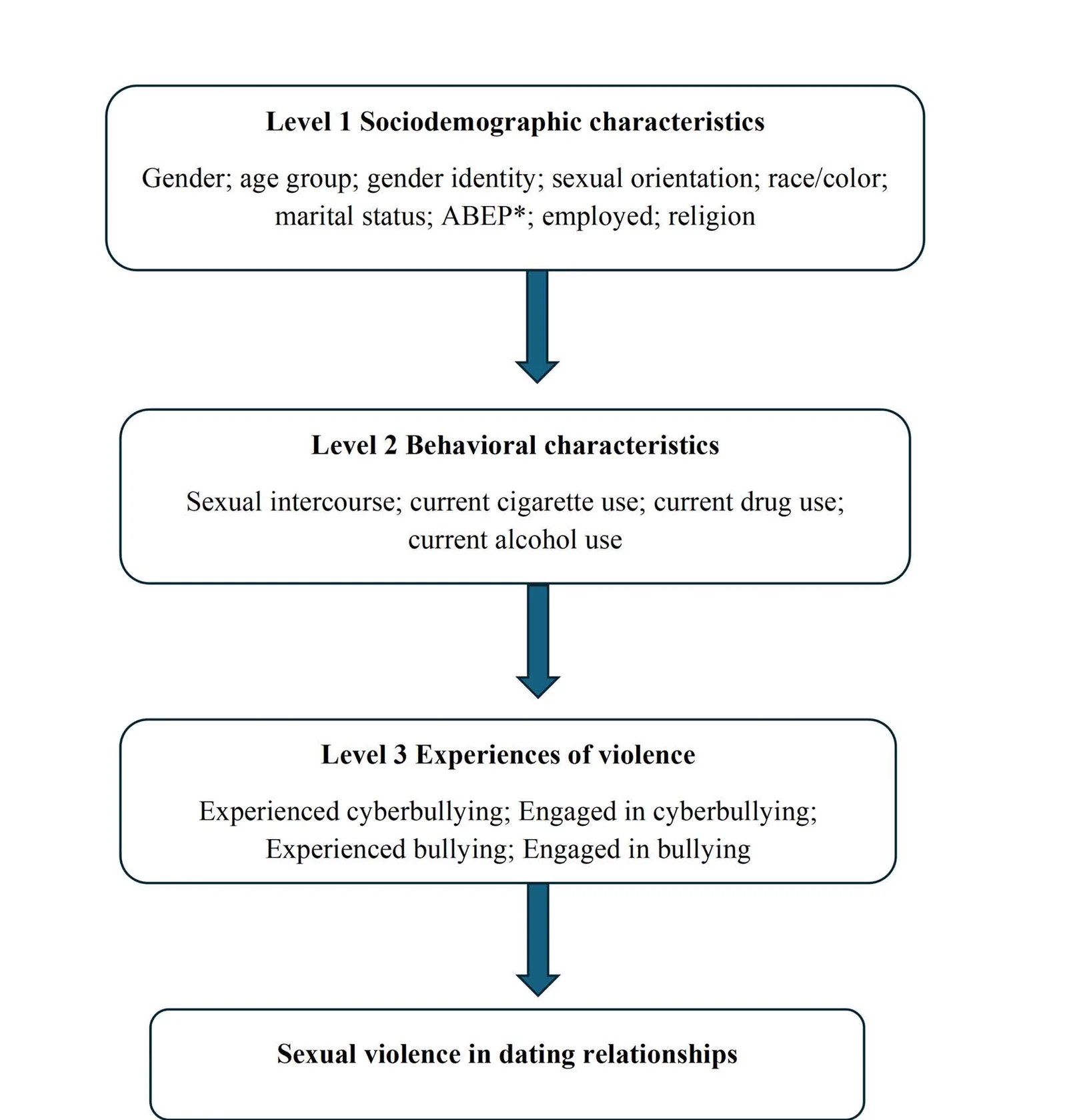
Level analysis model: flowchart

*ABEP = *Associação Brasileira de Empresas de Pesquisa*

The results were presented as adjusted odds ratios (ORa) and 95% confidence intervals (95% CI). The analyses were performed in Stata 17.0 to ensure robust identification of associated factors.

### Ethical considerations

In accordance with ethical considerations, the study was submitted to the Ethics and Research Committee of the Federal University of Espírito Santo and was carried out after approval and registration under opinion 5,900,370.

## Results

Table 1 shows the prevalence of sexual violence in dating relationships among high school adolescents. It should be noted that of the 1,238 adolescents who participated in the study, 7.8% (95% CI: 6.4-9.4) reported having experienced sexual violence in a dating relationship. When analyzing the results by gender, 9.3% (95% CI: 7.4-11.5) of female adolescents reported experiencing this type of violence, while among male adolescents, the prevalence was 5.1% (95% CI: 3.4-7.6).

**Table 1:** Prevalence of sexual violence in dating relationships among high school adolescents in the Metropolitan Region of Vitória, ES, Brazil, 2023

	General sample			Female			Male		
	N*	%^†^	95%CI^‡^	N*	%^†^	95%CI^‡^	N*	%^†^	95%CI^‡^
Sexual violence in dating relationships (n=1,238)									
Yes	96	7.8	6.4-9.4	73	9.3	7.4-11.5	23	5.1	3.4-7.6
No	1142	92.3	90.6-93.6	715	90.7	88.5-92.6	427	94.9	92.4-96.6

*N = Absolute frequency; ^†^% = Relative frequency; ^‡^95%CI = 95% Confidence Interval

Table 2 shows that there is a relationship between sexual violence in dating and the variables: gender, heterosexuality, religion, sexual intercourse at some point in life, current drug use, and current alcohol use, as well as among those who have suffered and practiced cyberbullying and suffered bullying (p<0.05).

**Table 2:** Distribution of sexual violence among high school adolescents in the Metropolitan Region of Vitória, ES, Brazil, 2023

Variables		Yes*	95%CI^†^	p-value^‡^
Sociodemographic				
**Gender (n=1,238)**				0.009
Female		73 (9.3)	7.4-11.5	
Male		23 (5.1)	3.4-7.6	
**Age group (n=1,238)**				0.277
≤15 years		14 (6.0)	3.6-9.9	
≥16 years		82 (8.2)	6.6-10.1	
**Cisgender (n=1,237)**				0.170
Yes		84 (7.4)	6.0-9.0	
No		11 (11.2)	6.3-19.2	
**Heterosexual (n=1,238)**				0.001
Yes		60 (6.4)	5.0-8.1	
No		36 (12.2)	8.9-16.5	
**Race/color (n=1,195)**				0.544
White (o)		34 (7.2)	5.2-10.0	
Black (o)		21 (9.6)	6.4-14.3	
Brown (o)		39 (7.7)	5.7-10.4	
**ABEP^§^ (n=1,237)**				0.067
A		9 (4.1)	2.1-7.7	
B		45 (8.1)	6.1-10.7	
C/D/E		42 (9.1)	6.8-12.1	
**Has a job/employment (n=1,238)**				0.442
No		62 (7.4)	5.8-9.3	
Yes		34 (8.6)	6.2-11.8	
**Religion (n=1,238)**				0.022
None		22 (7.1)	4.7-10.6	
Catholic		16 (5.3)	3.3-8.5	
Evangelical		42 (8.1)	6.0-10.8	
Others		16 (14.4)	9.0-22.3	
**Behavioral**				
**Sexual intercourse at some point in life (n=1,238)**				0.001
No		22 (4.6)	3.0-6.9	
Yes		74 (9.7)	7.8-12.1	
**Current cigarette use (n=402)**				0.220
No		35 (10.4)	7.5-14.1	
Yes		10 (15.6)	8.6-26.7	
**Current drug use (n=1,238)**				<0.001
No		71 (6.5)	5.2-8.2	
Yes		25 (16.7)	11.5-23.5	
**Current alcohol use (n=1,238)**				0.002
No		46 (5.9)	4.5-7.8	
Yes		50 (10.8)	8.3-14.0	
**Experiences of Violence**				
**They have experienced cyberbullying at some point in life (n=1,238)**				0.001
No		80 (7.0)	5.7-8.7	
Yes		16 (16.0)	10.0-24.5	
**They have engaged in cyberbullying in life (n=1,238)**				0.018
No		86 (7.3)	6.0-9.0	
Yes		10 (15.4)	8.5-26.3	
**They have been bullied in life (n=1,171)**				<0.001
No		22 (4.0)	2.6-5.9	
Yes		68 (11.1)	8.8-13.8	
**They have bullied someone in life (n=1,238)**				0.165
No		77 (7.3)	5.9-9.1	
Yes		19 (10.3)	6.6-15.5	

*Yes = Absolute frequency and relative frequency; ^†^95% CI = 95% confidence interval; ^‡^P-value = Significance level; ^§^ABEP = Brazilian Association of Research Companies

Table 3 shows that non-heterosexual adolescents were approximately twice as likely to experience sexual violence in dating relationships (ORa: 2.05; 95% CI: 1.32-3.16). Similarly, those who had had sexual intercourse were approximately twice as likely to experience sexual abuse (ORa: 1.91; 95% CI: 1.15-3.16). Adolescents who reported current drug use were 2.19 times more likely to experience sexual abuse by their partner (95% CI: 1.31-3.66). Another finding was related to those who had been bullied (ORa: 2.72; 95% CI: 1.63-4.52) and cyberbullied (ORa: 1.97; 95% CI: 1.07-3.64), which significantly increased their chances of experiencing sexual violence in dating relationships.

**Table 3:** Crude and adjusted analysis of sexual violence in dating relationships among high school adolescents in the Metropolitan Region of Vitória, ES, Brazil, 2023

Variables	OR*	95% CI^†^	P-value^‡^	ORa^§^	95% CI^†^	P-value^‡^
Sociodemographic						
**Gender (n=1.238)**			0.010			0.093
Female	1.90	1.17-3.07		1.55	0.93-2.58	
Male	1			1		
**Age group (n=1.238)**			0.279			-
≤15 years	1			-		
≥16 years	1.38	0.77-2.48		-	-	
**Cisgender (n=1.237)**			0.173			0.778
Yes	1			1		
No	1.59	0.82-3.09		1.11	0.54-2.26	
**Heterosexual (n=1.238)**			0.001			0.001
Yes	1			1.0		
No	2.05	1.32-3.16		2.05	1.32-3.16	
**Race/color (n=1.195)**			0.545			-
White (o)	1			-	-	
Black (o)	1.37	0.77-2.42		-	-	
Brown (o)	1.07	0.66-1.72		-	-	
**ABEP^||^ (n=1.237)**			0.076			0.063
A	1			1		
B	2.06	0.99-4.30		2.18	1.04-4.55	
C/D/E	2.35	1.12-4.92		2.42	1.15-5.08	
**Has a job/employment (n=1.238)**			0.443			-
No	1			-	-	
Yes	1.19	0.77-1.84		-	-	
**Religion (n=1.238)**			0.027			0.075
None	1			1		
Catholic	0.74	0.38-1.43		0.96	0.48-1.90	
Evangelical	1.15	0.67-1.97		1.44	0.82-2.54	
Others	2.20	1.11-4.36		2.22	1.10-4.46	
**Behavioral**						
**Sexual intercourse at some point** **in life (n=1.238)**			0.001			0.012
No	1			1		
Yes	2.24	1.37-3.65		1.91	1.15-3.16	
**Current cigarette use (n=402)**			0.224			-
No	1			-	-	
Yes	1.60	0.75-3.43		-	-	
**Current drug use (n=1.238)**			<0.001			0.003
No	1			1		
Yes	2.86	1.75-4.69		2.19	1.31-3.66	
**Current alcohol use (n=1.238)**			0.002			0.405
No	1			1		
Yes	1.93	1.27-2.93		1.22	0.76-1.96	
**Experiences of Violence**						
**They have experienced** **cyberbullying at some point in life** **(n=1.238)**			0.002			0.029
No	1			1		
Yes	2.52	1.41-4.50		1.97	1.07-3.64	
**They have engaged** **in cyberbullying in life (n=1.238)**			0.021			0.219
No	1			1		
Yes	2.30	1.13-4.67		1.63	0.75-3.57	
**They have been bullied in life** **(n=1.171)**			<0.001			<0.001
No	1			1		
Yes	3.03	1.85-4.97		2.72	1.63-4.52	
**They have bullied someone in life** **(n=1.238)**			0.167			0.568
No	1			1		
Yes	1.45	0.86-2.46		1.19	0.66-2.15	

*OR = Crude Odds Ratio; ^†^95% CI = 95% Confidence Interval; ^‡^P-value = Significance level; ^§^ORa = Adjusted Odds Ratio; ^||^ABEP = Brazilian Association of Research Companies

## Discussion

The overall prevalence of sexual violence in dating relationships among high school adolescents found in this study was 7.8% (95% CI: 6.4-9.4), indicating that a considerable proportion of adolescents experience sexual violence in their romantic relationships, which is a worrying reality considering the impacts of sexual violence at this stage of development.

In the United States, a survey conducted by the Centers for Disease Control and Prevention (CDC) found that approximately 7.5% of adolescents (95% CI: 6.4-8.7) had experienced sexual violence in dating relationships^10^. In Brazil, data from the National School Health Survey (PeNSE, in Portuguese) indicate that approximately 4% of adolescents reported having been forced to have sex, with their boyfriend or girlfriend or ex-boyfriend or ex-girlfriend being the main perpetrator^11^.

For girls, the prevalence of sexual violence in dating relationships was 9.3% (95% CI: 7.4-11.5) and 5.1% (95% CI: 3.4-7.6) for boys. A study on sexual violence among schoolchildren in Brazil, using data from the 2019 national school health survey, showed a prevalence of sexual violence in dating relationships of 8.8% (95% CI: 8.4-9.3) among females and 3.7% (95% CI: 3.3-4.0) among males^12^. In the United States, the CDC identified a significant gender disparity in dating violence, with 11.3% of girls compared to 3.5% of boys, indicating that female adolescents are three times more at risk^13^. It is a fact that girls are more vulnerable to this type of violence due to factors such as gender inequality, socialization based on female submission, and cultural norms that tolerate abusive behavior by romantic partners^14^.

This study indicated that non-heterosexual adolescents were approximately twice as likely to experience sexual violence in dating relationships (ORa: 2.05; 95% CI: 1.32-3.16). Similarly, data from the 2021 Youth Risk Behavior Survey, conducted in the United States, show that adolescents who identify as LGB reported experiences of sexual violence in dating relationships at a higher rate than their heterosexual peers^15^. Specifically, 17% of LGBTQ+ adolescents reported having been forced to have sex, compared to 6% of heterosexual adolescents^10^. This evidence reveals that belonging to sexual minorities represents an additional risk factor for dating violence, especially sexual violence. LGBTQIA+ adolescents are more exposed to various forms of violence and vulnerability due to structural discrimination, social stigma, and family rejection, which contributes to the invisibility and underreporting of these cases^16^.

This violence, which is often normalized or overlooked, occurs not only in relationships, but also in institutions such as schools and health services, which are not always prepared to welcome and protect these populations^17^. In addition, the lack of recognition of gender identity and the invalidation of sexual orientation by families intensify psychological distress, increasing the risk of depression, anxiety, substance use, and suicidal ideation^18^.

Furthermore, the results of this study indicate that adolescents who have already begun their sexual lives are more likely to suffer sexual violence in the context of dating (ORa: 1.91; 95% CI: 1.15-3.16). This finding is consistent with recent literature, which associates early or unplanned sexual initiation with a series of social and behavioral vulnerabilities that may favor situations of abuse. According to the study, adolescents who began their sexual lives at age 13 or younger had a higher prevalence of risky sexual behaviors, including less condom use and greater exposure to sexual violence^19^.

Current drug use was also associated with a 2.19-fold increase in the likelihood of suffering sexual abuse by a partner (95% CI: 1.31-3.66). A literature review highlights that dating violence among adolescents is a relevant public health issue, influenced by factors such as psychoactive substance use, which can contribute to increased aggressive behavior in relationships among this age group^20^. Additionally, a study conducted in Nicaragua with 1,799 adolescents revealed that the prevalence of dating violence was associated with the use of psychoactive substances such as alcohol, traditional cigarettes, marijuana, electronic cigarettes, and external behaviors such as aggression, disobedience, antisocial conduct, and other disruptive behaviors. The results indicate that adolescents who consume psychoactive substances are more likely to engage in abusive relationships^20^.

It has been observed that experiencing bullying is associated with greater vulnerability among adolescents to experiences of violence and interpersonal relationships marked by coercion and abuse, indicating the possibility of victimization being transferred between different social contexts. Population-based evidence shows that adolescents exposed to bullying are more prone to risky behaviors and unhealthy interpersonal relationships, increasing their susceptibility to abusive experiences throughout their development^21^ ^-^ ^22^. Similarly, a study identified the relationship between bullying and violence among adolescent couples, highlighting that those who have experienced aggression or humiliation in the school environment are more likely to tolerate abusive behavior in their relationships^19^.

The limitations of this study include its cross-sectional design, which captures a single point in time, precluding robust causal analyses and leaving it susceptible to recall bias in the data collection instruments. However, the study makes a substantial contribution by pioneering the mapping of patterns of dating violence among young people in a major Brazilian city, providing representative data for this specific population.

## Conclusion

The prevalence of 7.8% of sexual violence in dating relationships identified among the adolescents participating in this study highlights the relevance and urgency of addressing this specific type of violence in youth romantic relationships. This data reinforces that sexual victimization is already present in early romantic experiences.

The present study demonstrated that sexual violence in adolescent dating relationships is significantly associated with factors such as current use of psychoactive substances, non-heterosexual adolescents, those who have had sexual intercourse at some point in their lives, and those who have suffered bullying and cyberbullying. These findings indicate that sexual victimization in the context of romantic relationships among adolescents occurs concurrently with other experiences of exposure to violence and risk behaviors, highlighting the importance of prevention strategies that consider the complexity of these associations and the coexistence of different forms of vulnerability during adolescence.

Such evidence is particularly relevant for health, education, and social work professionals, as well as policymakers, as it identifies groups and contexts that warrant greater attention in prevention and protection efforts for adolescent romantic relationships. Recognition of these associated factors can inform the development of intersectoral strategies that promote safer, more equitable, and respectful relationships among adolescents.

Therefore, the results of this study reiterate the importance of developing and implementing policies and programs to prevent sexual violence in dating relationships, with an emphasis on reducing identified associated factors and promoting healthy emotional relationships among adolescents.

## Data Availability

All data generated or analysed during this study are included in this published article.
