# Supplementary material for: Sexual violence in dating relationships among high school adolescents: a school-based study
Source: Rev Lat Am Enfermagem. 2026 Oct 9;34:e4918. doi: 10.1590/1518-8345.8316.4918 (PMC13652529; doi:10.1590/1518-8345.8316.4918)
Supplement: Translation

# Translation: Violência sexual no namoro entre adolescentes do ensino médio: um estudo de base escolar^*^

**DOI:** 10.1590/1518-8345.8316.4919

**Authors:** Isaura Barros Alves Pinto, Rita Mafalda Costa Francisco, Franciele Marabotti Costa Leite

**Affiliations:** 1 Universidade Federal do Espírito Santo, Departamento de Enfermagem, Vitória, ES, Brasil.; 2 Instituto de Medicina Social, Universidade do Estado do Rio de Janeiro, Rio de Janeiro, RJ, Brasil.; 3 Departamento do Programa Nacional de Imunizações, Ministério da Saúde, Brasília, DF, Brasil.; 4 Universidade Católica Portuguesa, Faculdade de Ciências Humanas, Lisboa, Portugal.

**Keywords:** Violência, Violência por Parceiro Íntimo, Saúde do Adolescente, Epidemiologia, Saúde Pública, Enfermagem.

## Abstract

**Objetivo:**

analisar a prevalência de violência sexual no namoro entre adolescentes do ensino médio e sua associação com as características socioeconômicas, comportamentais e experiências de violências.

**Método:**

estudo transversal e analítico realizado com 1.238 adolescentes de 14 a 19 anos, matriculados em escolas públicas e privadas, que estavam em relacionamento afetivo no momento da pesquisa. A amostragem foi por conglomerados e estratificação.** **

**Resultados:**

dos 1.238 adolescentes em relacionamento afetivo, 7,8% (IC 95%: 6,4-9,4) relataram já ter sofrido violência sexual no namoro. A prevalência foi de 9,3% entre meninas e 5,1% entre meninos. Observa-se maiores chances de violência sexual no namoro entre os adolescentes não heterossexuais, os que tiveram relação sexual alguma vez na vida, os que usam drogas e os que sofreram *bullying* e *cyberbullying* (p<0,05).** **

**Conclusão:**

é fundamental que os achados contribuam para o fortalecimento de campanhas educativas nas escolas, que abordem não apenas os comportamentos abusivos, mas também a importância do consentimento, do respeito mútuo e da construção de vínculos afetivos seguros entre os adolescentes.

## Destaques

**(1)** Prevalência de 7,8% de violência sexual no namoro adolescente.

**(2)** Achados reforçam a urgência de enfrentar a violência nas relações juvenis.

**(3)** Vitimização sexual afeta adolescentes já nos primeiros relacionamentos amorosos.

**(4)** Violência sexual no namoro se associa a drogas, *bullying*, *cyberbullying*, relação sexual alguma vez na vida e não heterossexuais.

**(5)** Parceria com saúde, escola e família favorece a escuta e prevenção do abuso.

## Introdução

A adolescência marca um período de transição no qual as relações interpessoais, especialmente amizades e namoros, assumem centralidade, enquanto os vínculos familiares se reconfiguram^1^. Nessa fase, a interação de fatores individuais e sociais pode favorecer a internalização de normas que naturalizam a violência sexual^2^ ^-^ ^3^.

A violência no namoro (VN), compreendida como qualquer forma de agressão física, emocional ou sexual por um parceiro íntimo^4^, frequentemente se manifesta como um mecanismo de controle e dominação. Dentre suas expressões, a violência sexual destaca-se por envolver coerção, assédio ou imposição de atos sexuais não consentidos, configurando-se como uma violação grave dos direitos humanos^5^. Estudos indicam que essa violência pode ocorrer em relacionamentos não conjugais, independentemente da orientação sexual^6^.

Dados epidemiológicos ilustram a magnitude do problema: pesquisa com adolescentes brasileiros^7^ identificou que 38% relataram experiências de violência sexual no namoro, incluindo pressão para atividades íntimas e divulgação não consensual de imagens. Essas vivências correlacionam-se com consequências como depressão, estresse pós-traumático e uso de substâncias^8^. Ademais, a normalização dessas agressões, muitas vezes enraizada em estereótipos de gênero, dificulta a identificação e a denúncia^3^.

Diante desse cenário, este estudo visa analisar a prevalência de violência sexual no namoro entre adolescentes do ensino médio e sua associação com as características socioeconômicas, comportamentais e experiências de violências.

## Método 

### Delineamento do estudo

Trata-se de um estudo transversal, analítico, realizado em escolas públicas e privadas, localizadas em municípios da Região Metropolitana da Grande Vitória (RMGV), no Estado do Espírito Santo. 

### Local

A Região Metropolitana da Grande Vitória (RMGV) é composta pelos municípios de Cariacica, Fundão, Guarapari, Serra, Viana, Vila Velha e Vitória. Essa região abriga aproximadamente 1.880.828 habitantes, distribuídos em uma área de 2.331 km²^9^.

### Período 

A coleta de dados ocorreu entre março e dezembro de 2023.

### População

A população-alvo do estudo foram adolescentes do ensino médio, de 14 a 19 anos, regularmente matriculados em escolas públicas ou privadas da RMGV que participaram a partir do aceite dos responsáveis através do Termo de Consentimento Livre e Esclarecido (TCLE) e a assinatura dos adolescentes pelo Termo de Assentimento Livre e Esclarecido (TALE), sendo esclarecidos acerca dos objetivos da pesquisa e seus eventuais riscos e benefícios. 

### Definição da amostra

A amostragem utilizou o percentual de 95% de confiança e erro máximo de 5%. Para cada estrato de município, o erro máximo foi de 10%, sendo a amostra mínima de 4.416 estudantes. Para o presente estudo, foram selecionados apenas os adolescentes que possuíam algum tipo de relacionamento no momento da entrevista, totalizando uma amostra de 1.238 adolescentes.

Antes da etapa de coleta de dados, as escolas sorteadas foram convidadas por e-mail para participarem de um seminário de apresentação da pesquisa e, posteriormente, foi feito contato por e-mail/telefone para o agendamento da coleta de dados. Importante destacar que, anteriormente ao trabalho de campo, foi realizado o recrutamento e treinamento da equipe, e foi realizado o teste-piloto no mês de fevereiro de 2023. A coleta de dados aconteceu de março a dezembro de 2023, por meio de *tablets*, onde o adolescente autorrespondia ao questionário estruturado no sistema REDCap (*Research Electronic Data Capture*).

### Variáveis do estudo

O desfecho no presente estudo foi a violência sexual no namoro, que foi dicotomizada (sim/não). A variável - violência sexual no namoro - foi considerada afirmativa se o adolescente tivesse vivenciado pelo menos uma das situações: “o(a) parceiro(a) fez carícias sem seu consentimento e vontade, te forçou a ter relação sexual quando você não queria, impediu o uso de método preservativo, induziu ao aborto ou qualquer outra questão que infringiu seus direitos sexuais e reprodutivos”. 

Quanto às variáveis independentes, foram analisadas as sociodemográficas: sexo (feminino/masculino); faixa etária (≤15 anos/≥16 anos); cisgênero (sim/não); heterossexual (sim/não); raça/cor (branca(o)/preta(o)/parda(o); A classificação econômica mensurada pelo Critério de Classificação Econômica da Associação Brasileira de Empresas de Pesquisa (ABEP), que classifica em classe A/B/C/D/E; possui trabalho/emprego (não/sim); e religião (nenhuma/católica/evangélica/outras). Foram também analisadas as seguintes variáveis comportamentais: relação sexual alguma vez na vida (não/sim); uso atual de cigarro (não/sim); uso atual de drogas (não/sim) e uso atual de álcool (não/sim), assim como as variáveis relacionadas às experiências de violências: sofreu *cyberbullying* alguma vez na vida (não/sim); praticou *cyberbullying* alguma vez na vida (não/sim); sofreu *bullying* alguma vez na vida (não/sim); praticou *bullying* alguma vez na vida (não/sim).

### Tratamento e análise dos dados

Para as análises estatísticas descritivas, foram calculadas frequências absolutas e relativas. Na análise bivariada, utilizou-se o teste Qui-quadrado de Pearson (χ2) conforme pressupostos, com seus respectivos intervalos de confiança de 95%. Para análise multivariada bruta e ajustada por Razão de Chances através de Regressão Logística para avaliar associações entre variáveis independentes e violência sexual no namoro, inicialmente, foi realizada análise descritiva e bivariada com cálculo do *Odds Ratio* (OR) bruto. Variáveis com p ≤0,20 foram incluídas no modelo multivariado, ajustado para fatores de confusão, e permaneceram no modelo o valor de p ≤ 0,05 (Figura 1). 

**Figura 1:** Modelo de Análise em níveis: fluxograma

*ABEP = Associação Brasileira de Empresas de Pesquisa

Os resultados foram apresentados como *Odds Ratio* ajustada (ORa) e intervalos de confiança de 95% (IC95%). As análises foram realizadas no *software* Stata 17.0, garantindo robustez na identificação dos fatores associados.

### Considerações éticas

Preservando as questões éticas, o estudo foi enviado ao Comitê de Ética e Pesquisa da Universidade Federal do Espírito Santo e foi executado após aprovação e registro sob o parecer 5.900.370.

## Resultados

A Tabela 1 mostra a prevalência de violência sexual no namoro entre os adolescentes do Ensino Médio. Nota-se que dos 1.238 adolescentes participantes da pesquisa, 7,8% (IC95%: 6,4-9,4) afirmaram já ter sofrido violência sexual no namoro. Ao analisar os resultados por sexo, 9,3% (IC95%: 7,4-11,5) das adolescentes do sexo feminino relataram a vivência desse tipo de violência, enquanto entre os adolescentes do sexo masculino a prevalência foi de 5,1% (IC95%: 3,4-7,6).

**Tabela 1:** Prevalência de violência sexual no namoro entre adolescentes do Ensino Médio da Região Metropolitana de Vitória, ES, Brasil, 2023

	Amostra geral			Feminino			Masculino		
	N*	%^†^	IC95%^‡^	N*	%^†^	IC95%^‡^	N*	%^†^	IC95%^‡^
Violência sexual no namoro (n=1.238)									
Sim	96	7,8	6,4-9,4	73	9,3	7,4-11,5	23	5,1	3,4-7,6
Não	1142	92,3	90,6-93,6	715	90,7	88,5-92,6	427	94,9	92,4-96,6

*N = Frequência absoluta; ^†^% = Frequência relativa; ^‡^IC95% = Intervalo de Confiança de 95%

A Tabela 2 mostra que há uma relação entre a violência sexual no namoro e as variáveis: sexo, heterossexual, religião, relação sexual alguma vez na vida, uso atual de drogas e uso atual de álcool, assim como entre aqueles que já sofreram e praticaram *cyberbullying* e sofreram *bullying* (p<0,05).

**Tabela 2:** Distribuição de violência sexual entre adolescentes do Ensino Médio da Região Metropolitana de Vitória, ES, Brasil, 2023

Variáveis	Sim*	IC95%^†^	p-valor^‡^
Sociodemográficas			
**Sexo (n=1.238)**			0,009
Feminino	73 (9,3)	7,4-11,5	
Masculino	23 (5,1)	3,4-7,6	
**Faixa etária (n=1.238)**			0,277
≤15 anos	14 (6,0)	3,6-9,9	
≥16 anos	82 (8,2)	6,6-10,1	
**Cisgênero (n=1.237)**			0,170
Sim	84 (7,4)	6,0-9,0	
Não	11 (11,2)	6,3-19,2	
**Heterossexual (n=1.238)**			0,001
Sim	60 (6,4)	5,0-8,1	
Não	36 (12,2)	8,9-16,5	
**Raça/cor (n=1.195)**			0,544
Branca (o)	34 (7,2)	5,2-10,0	
Preta (o)	21 (9,6)	6,4-14,3	
Parda (o)	39 (7,7)	5,7-10,4	
**ABEP^§^ (n=1.237)**			0,067
A	9 (4,1)	2,1-7,7	
B	45 (8,1)	6,1-10,7	
C/D/E	42 (9,1)	6,8-12,1	
**Possui trabalho/emprego (n=1.238)**			0,442
Não	62 (7,4)	5,8-9,3	
Sim	34 (8,6)	6,2-11,8	
**Religião (n=1.238)**			0,022
Nenhuma	22 (7,1)	4,7-10,6	
Católica	16 (5,3)	3,3-8,5	
Evangélica	42 (8,1)	6,0-10,8	
Outras	16 (14,4)	9,0-22,3	
**Comportamentais**			
**Relação sexual alguma vez na vida (n=1.238)**			0,001
Não	22 (4,6)	3,0-6,9	
Sim	74 (9,7)	7,8-12,1	
**Uso de cigarro atual (n=402)**			0,220
Não	35 (10,4)	7,5-14,1	
Sim	10 (15,6)	8,6-26,7	
**Uso de drogas atual (n=1.238)**			<0,001
Não	71 (6,5)	5,2-8,2	
Sim	25 (16,7)	11,5-23,5	
**Uso de álcool atual (n=1.238)**			0,002
Não	46 (5,9)	4,5-7,8	
Sim	50 (10,8)	8,3-14,0	
**Experiências de Violências**			
**Sofreu *cyberbullying* alguma vez na vida (n=1.238)**			0,001
Não	80 (7,0)	5,7-8,7	
Sim	16 (16,0)	10,0-24,5	
**Praticou *cyberbullying* alguma vez na vida (n=1.238)**			0,018
Não	86 (7,3)	6,0-9,0	
Sim	10 (15,4)	8,5-26,3	
**Sofreu *bullying* alguma vez na vida (n=1.171)**			<0,001
Não	22 (4,0)	2,6-5,9	
Sim	68 (11,1)	8,8-13,8	
**Praticou *bullying* alguma vez na vida (n=1.238)**			0,165
Não	77 (7,3)	5,9-9,1	
Sim	19 (10,3)	6,6-15,5	

*Sim = Frequência absoluta e frequência relativa; ^†^IC95% = Intervalo de Confiança de 95%; ^‡^P-valor = Nível de significância; ^§^ABEP = Associação Brasileira de Empresas de Pesquisa

A Tabela 3 mostra que adolescentes não heterossexuais apresentaram cerca de duas vezes mais chance de violência sexual no namoro (ORa: 2,05; IC95%: 1,32-3,16). No mesmo sentido, aqueles que já tiveram relação sexual alguma vez na vida apresentaram aproximadamente duas vezes mais chances de abusos sexuais (ORa: 1,91; IC95%: 1,15-3,16). Adolescentes que relataram o uso de drogas atualmente tiveram 2,19 vezes mais chance de abuso sexual pelo parceiro(a) (IC95%: 1,31-3,66). Outro resultado encontrado foi em relação àqueles que sofreram *bullying* (ORa: 2,72; IC95%: 1,63-4,52) e *cyberbullying* (ORa: 1,97; IC95%: 1,07-3,64), que aumentou significativamente as chances de sofrerem a violência sexual no namoro.

**Tabela 3:** Análise bruta e ajustada da violência sexual no namoro entre adolescentes do Ensino Médio da Região Metropolitana de Vitória, ES, Brasil, 2023

Variáveis	OR*	IC95%^†^	P-valor^‡^	ORa^§^	IC95%^†^	P-valor^‡^
Sociodemográficas						
**Sexo (n=1.238)**			0,010			0,093
Feminino	1,90	1,17-3,07		1,55	0,93-2,58	
Masculino	1			1		
**Faixa etária (n=1.238)**			0,279			-
≤15 anos	1			-		
≥16 anos	1,38	0,77-2,48		-	-	
**Cisgênero (n- 1.237)**			0,173			0,778
Sim	1			1		
Não	1,59	0,82-3,09		1,11	0,54-2,26	
**Heterossexual (n=1.238)**			0,001			0,001
Sim	1			1,0		
Não	2,05	1,32-3,16		2,05	1,32-3,16	
**Raça/cor (n=1.195)**			0,545			-
Branca (o)	1			-	-	
Preta (o)	1,37	0,77-2,42		-	-	
Parda (o)	1,07	0,66-1,72		-	-	
**ABEP^||^ (n=1.237)**			0,076			0,063
A	1			1		
B	2,06	0,99-4,30		2,18	1,04-4,55	
C/D/E	2,35	1,12-4,92		2,42	1,15-5,08	
**Possui trabalho/emprego (n=1.238)**			0,443			-
Não	1			-	-	
Sim	1,19	0,77-1,84		-	-	
**Religião (n=1.238)**			0,027			0,075
Nenhuma	1			1		
Católica	0,74	0,38-1,43		0,96	0,48-1,90	
Evangélica	1,15	0,67-1,97		1,44	0,82-2,54	
Outras	2,20	1,11-4,36		2,22	1,10-4,46	
Comportamentais						
**Relação sexual alguma vez na vida (n=1.238)**			0,001			0,012
Não	1			1		
Sim	2,24	1,37-3,65		1,91	1,15-3,16	
**Uso de cigarro atual (n=402)**			0,224			-
Não	1			-	-	
Sim	1,60	0,75-3,43		-	-	
**Uso de drogas atual (n=1.238)**			<0,001			0,003
Não	1			1		
Sim	2,86	1,75-4,69		2,19	1,31-3,66	
**Uso de álcool atual (n=1.238)**			0,002			0,405
Não	1			1		
Sim	1,93	1,27-2,93		1,22	0,76-1,96	
Experiências de Violências						
**Sofreu *cyberbullying* alguma vez na vida (n=1.238)**			0,002			0,029
Não	1			1		
Sim	2,52	1,41-4,50		1,97	1,07-3,64	
**Praticou *cyberbullying* alguma vez na vida (n=1.238)**			0,021			0,219
Não	1			1		
Sim	2,30	1,13-4,67		1,63	0,75-3,57	
**Sofreu *bullying* alguma vez na vida (n=1.171)**			<0,001			<0,001
Não	1			1		
Sim	3,03	1,85-4,97		2,72	1,63-4,52	
**Praticou *bullying* alguma vez na vida (n=1.238)**			0,167			0,568
Não	1			1		
Sim	1,45	0,86-2,46		1,19	0,66-2,15	

*OR = *Odds Ratio* bruto; ^†^IC95% = Intervalo de Confiança de 95%; ^‡^P-valor = Nível de significância; ^§^ORa = *Odds Ratio* Ajustado; ^||^ABEP = Associação Brasileira de Empresas de Pesquisa

## Discussão

A prevalência geral de violência sexual no namoro entre os adolescentes do ensino médio, encontrada neste estudo, foi de 7,8% (IC95%: 6,4-9,4), o que indica que uma parcela considerável de adolescentes vivencia a violência sexual em suas relações afetivas, e representa uma realidade preocupante, considerando os impactos da violência sexual nessa fase do desenvolvimento. Nos Estados Unidos, uma pesquisa conduzida pelo *Centers for Disease Control and Prevention* (CDC) identificou que cerca de 7,5% dos adolescentes (IC95%: 6,4-8,7) sofreram violência sexual no namoro^10^. No Brasil, dados da Pesquisa Nacional de Saúde do Escolar (PeNSE) apontam que aproximadamente 4% dos adolescentes relataram ter sido forçados a manter relações sexuais, sendo o(a) namorado(a) ou ex-namorado(a) o principal agressor^11^.

Para as meninas, a prevalência de violência sexual no namoro foi de 9,3% (IC95%: 7,4-11,5) e 5,1% (IC95%: 3,4-7,6) para os meninos. Um estudo sobre a violência sexual em escolares no Brasil, com dados da pesquisa nacional de saúde do escolar de 2019, mostrou no sexo feminino uma prevalência de violência sexual no namoro de 8,8% (IC95%:8,4-9,3) e no sexo masculino de 3,7% (IC95%: 3,3-4,0)^12^. Nos Estados Unidos, o CDC identificou disparidade significativa por gênero na violência sexual no namoro, 11,3% das meninas contra 3,5% dos meninos, indicando que adolescentes do sexo feminino têm risco três vezes maior^13^. É fato que meninas apresentam maior vulnerabilidade a esse tipo de violência, em razão de fatores como desigualdade de gênero, socialização baseada na submissão feminina e normas culturais que toleram comportamentos abusivos por parte de parceiros afetivos^14^.

O presente estudo evidenciou que adolescentes não heterossexuais apresentaram cerca de duas vezes mais chance de sofrer violência sexual no namoro (ORa: 2,05; IC95%: 1,32-3,16). Em consonância, os dados do *Youth Risk Behavior Survey* de 2021, realizado nos Estados Unidos, evidenciam que adolescentes que se identificam como LGB relataram, em maior proporção do que seus pares heterossexuais, experiências de violência sexual no namoro^15^. Especificamente, 17% dos adolescentes LGBTQ+ afirmaram já ter sido forçados a manter relações sexuais, em comparação com 6% dos adolescentes heterossexuais^10^. Essas evidências revelam que o pertencimento a minorias sexuais representa um fator de risco adicional para a violência no namoro, especialmente a sexual. Adolescentes LGBTQIA+ estão mais expostos a diversas formas de violência e vulnerabilidade devido à discriminação estrutural, ao estigma social e à rejeição familiar, o que contribui para a invisibilidade e subnotificação desses casos^16^.

Essas violências, frequentemente naturalizadas ou negligenciadas, ocorrem não apenas no âmbito relacional, mas também nas instituições, como escolas e serviços de saúde, que nem sempre estão preparados para acolher e proteger essas populações^17^. Além disso, a ausência de reconhecimento da identidade de gênero e a invalidação da orientação sexual por parte das famílias intensificam o sofrimento psíquico, elevando o risco de depressão, ansiedade, uso de substâncias e ideação suicida^18^. 

Além disso, os resultados do presente estudo indicam que adolescentes que já iniciaram a vida sexual apresentam maior chance de sofrer violência sexual no contexto do namoro (ORa: 1,91; IC95%: 1,15-3,16). Esse achado é coerente com a literatura recente, que associa a iniciação sexual precoce ou não planejada a uma série de vulnerabilidades sociais e comportamentais que podem favorecer situações de abuso. Segundo o estudo, adolescentes que iniciaram a vida sexual com 13 anos ou menos apresentaram maior prevalência de comportamentos sexuais de risco, incluindo menor uso de preservativos e maior exposição à violência sexual^19^.

O uso atual de drogas também esteve associado a um aumento de 2,19 vezes na chance de sofrer abuso sexual por parte do parceiro ou parceira (IC95%: 1,31-3,66). Em uma revisão de literatura, destacam que a violência no namoro entre adolescentes representa uma questão relevante para a saúde pública, sendo influenciada por fatores como o consumo de substâncias psicoativas, que podem contribuir para o aumento de comportamentos agressivos nos relacionamentos dessa faixa etária^20^. Adicionalmente, um estudo conduzido na Nicarágua com 1.799 adolescentes revelou que a prevalência de violência no namoro esteve associada ao uso de substâncias psicoativas como álcool, cigarros tradicionais, maconha, cigarros eletrônicos, e a comportamentos externos como agressividade, desobediência, condutas antissociais, e outros comportamentos disruptivos. Os resultados indicam que adolescentes que consomem substâncias psicoativas apresentam maiores chances de se envolver em relacionamentos abusivos^20^.

Observou-se que a vivência de *bullying* está associada à maior vulnerabilidade dos adolescentes às experiências de violências e relações interpessoais marcadas por coerção e abuso, indicando a possibilidade de transferência da vitimização entre diferentes contextos sociais. Evidências de base populacional mostram que adolescentes expostos ao *bullying* apresentam maior propensão a comportamentos de risco e a relações interpessoais não saudáveis, ampliando a suscetibilidade a experiências abusivas ao longo do desenvolvimento^21^ ^-^ ^22^. De forma análoga, um estudo identificou a relação entre o bullying e a violência entre casais adolescentes, destacando que aqueles que passaram por situações de agressão ou humilhação no ambiente escolar apresentam maior tendência a tolerar comportamentos abusivos em seus relacionamentos^19^. 

A presente investigação apresenta como limitações a natureza transversal da pesquisa, ao capturar um único momento temporal, impedindo análises causais robustas, ficando também suscetível a vieses de recordação nos instrumentos de coleta. Entretanto, a pesquisa oferece contribuições substanciais ao mapear pioneiramente os padrões de violência no namoro entre jovens de uma importante metrópole brasileira, fornecendo dados representativos para esta população específica.

## Conclusão

A prevalência de 7,8% de violência sexual no namoro identificada entre os adolescentes participantes deste estudo evidencia a relevância e a urgência do enfrentamento desse tipo específico de violência nas relações afetivas juvenis. Esse dado reforça que a vitimização sexual está presente já nas primeiras experiências amorosas.

O presente estudo demonstrou que a violência sexual no namoro entre adolescentes está significativamente associada a fatores como o uso atual de substâncias psicoativas, a adolescentes não heterossexuais, aos que tiveram relação sexual alguma vez na vida, e os que sofreram *bullying* e *cyberbullying*. Esses achados indicam que a vitimização sexual no contexto das relações afetivas entre adolescentes ocorre de forma concomitante a outras experiências de exposição à violência e a comportamentos de risco, ressaltando a importância de estratégias de prevenção que considerem a complexidade dessas associações e a coexistência de diferentes formas de vulnerabilidade no período da adolescência.

Tais evidências são particularmente relevantes para profissionais da saúde, educação e assistência social, bem como para formuladores de políticas públicas, ao apontarem grupos e contextos que demandam maior atenção em ações de prevenção e proteção voltadas às relações afetivas juvenis. O reconhecimento desses fatores associados pode contribuir para o direcionamento de estratégias intersetoriais que promovam relações mais seguras, igualitárias e respeitosas entre adolescentes.

Portanto, os resultados deste estudo reiteram a importância do desenvolvimento e da implementação de políticas e programas de prevenção da violência sexual no namoro, com ênfase na redução de fatores associados identificados e na promoção de relações afetivas saudáveis entre adolescentes.

## Data Availability

Todos os dados gerados ou analisados durante este estudo estão incluídos neste artigo publicado.

# Translation: Violencia sexual en el noviazgo entre adolescentes de la educación secundaria: un estudio de base escolar^*^

**DOI:** 10.1590/1518-8345.8316.4917

**Authors:** Isaura Barros Alves Pinto, Rita Mafalda Costa Francisco, Franciele Marabotti Costa Leite

**Affiliations:** 1 Universidade Federal do Espírito Santo, Departamento de Enfermagem, Vitória, ES, Brasil.; 2 Instituto de Medicina Social, Universidade do Estado do Rio de Janeiro, Rio de Janeiro, RJ, Brasil.; 3 Departamento do Programa Nacional de Imunizações, Ministério da Saúde, Brasília, DF, Brasil.; 4 Universidade Católica Portuguesa, Faculdade de Ciências Humanas, Lisboa, Portugal.

**Keywords:** Violencia, Violencia de Pareja, Salud del Adolescente, Epidemiología, Salud Pública, Enfermería.

## Abstract

**Objetivo:**

analizar la prevalencia de violencia sexual en el noviazgo entre adolescentes de la educación secundaria y su asociación con las características socioeconómicas, comportamentales y experiencias de violencias.

**Método:**

estudio transversal y analítico realizado con 1.238 adolescentes entre los 14 y 19 años, matriculados en escuelas públicas y privadas, que mantenían una relación afectiva en el momento de la investigación. El muestreo se realizó por racimos y estratificación.** **

**Resultados:**

de los 1.238 adolescentes en una relación afectiva, el 7,8% (IC 95%: 6,4-9,4) reportó haber sufrido violencia sexual en el noviazgo. La prevalencia fue del 9,3% entre las niñas y del 5,1% entre los niños. Se observaron mayores probabilidades de violencia sexual en el noviazgo entre adolescentes no heterosexuales, aquellos que tuvieron relaciones sexuales al menos una vez en su vida, quienes consumieron drogas y quienes sufrieron *bullying* y *cyberbullying* (“ciberacoso”) (p<0,05).

**Conclusión:**

es esencial que los hallazgos contribuyan al fortalecimiento de las campañas educativas en las escuelas, que aborden no solo los comportamientos abusivos, sino también la importancia del consentimiento, el respeto mutuo y la construcción de vínculos afectivos seguros entre los adolescentes.

## Destacados

**(1)** Prevalencia de 7,8% de violencia sexual en el noviazgo adolescente.

**(2)** Los hallazgos refuerzan la urgencia de afrontar la violencia en las relaciones juveniles.

**(3)** La victimización sexual afecta adolescentes que ya están en sus primeras relaciones amorosas.

**(4)** Violencia sexual en el noviazgo está asociada con las drogas, *bullying* (“acoso escolar”), *cyberbullying*, sexo alguna vez en la vida y no heterosexuales.

**(5)** Colaboración con la salud, la escuela y la familia favorece la escucha y la prevención del abuso.

## Introducción

La adolescencia marca un periodo de transición en el que las relaciones interpersonales, especialmente las amistades y los noviazgos, asumen centralidad, mientras que los lazos familiares se reconfiguran^1^. En esta fase, la interacción de factores individuales y sociales puede favorecer la internalización de normas que naturalizan la violencia sexual^2^ ^-^ ^3^.

La violencia en el noviazgo (VN), entendida como cualquier forma de agresión física, emocional o sexual por parte de una pareja íntima^4^, suele manifestarse como un mecanismo de control y dominación. Entre sus expresiones, la violencia sexual destaca por implicar coacción, acoso o imposición de actos sexuales no consentidos, configurándose como una grave violación de los derechos humanos^5^. Los estudios indican que esta violencia puede ocurrir en relaciones no matrimoniales, independientemente de la orientación sexual^6^.

Los datos epidemiológicos ilustran la magnitud del problema: una encuesta con adolescentes brasileños^7^ identificó que el 38% reportó experiencias de violencia sexual en el noviazgo, incluyendo presión para actividades íntimas y difusión no consensuada de imágenes. Estas experiencias se correlacionan con consecuencias como la depresión, el estrés postraumático y el uso de sustancias^8^. Además, la normalización de estas agresiones, a menudo arraigadas en estereotipos de género, dificulta identificarlas y denunciarlas^3^.

En vista de este escenario, este estudio pretende analizar la prevalencia de la violencia sexual en el noviazgo entre adolescentes de secundaria y su asociación con características socioeconómicas y conductuales y experiencias de violencias.

## Método 

### Diseño del estudio

Se trata de un estudio transversal y analítico, realizado en escuelas públicas y privadas, ubicadas en municipios de la Región Metropolitana de la Grande Vitória (RMGV), en el Estado de Espírito Santo.

### Local

La Región Metropolitana de la Grande Vitória (RMGV) está compuesta por los municipios de Cariacica, Fundão, Guarapari, Serra, Viana, Vila Velha y Vitória. Esta región alberga aproximadamente 1.880.828 habitantes, distribuidos en un área de 2.331 km²^9^.

### Periodo 

La recopilación de datos tuvo lugar entre marzo y diciembre de 2023.

### Población

La población objetivo del estudio eran adolescentes de educación secundaria, entre los 14 y 19 años, matriculados regularmente en escuelas públicas o privadas en el RMGV, que participaban tras la aceptación de los padres o tutores mediante el Formulario de Consentimiento Libre, Previo e Informado (CLPI) y la firma de los adolescentes mediante el Formulario de Asentimiento Libre e Informado (FALI), siendo informados sobre los objetivos de la investigación y sus posibles riesgos y beneficios.

### Definición de la muestra

El muestreo utilizó un porcentaje de confianza del 95% y un error máximo del 5%. Para cada estrato municipal, el error máximo fue del 10%, con una muestra mínima de 4.416 estudiantes. En el presente estudio, solo se seleccionaron adolescentes que tenían algún tipo de relación en el momento de la entrevista, sumando una muestra de 1.238 adolescentes.

Antes de la fase de recopilación de datos, las escuelas seleccionadas fueron invitadas por correo electrónico a participar en un seminario para presentar la investigación y, a partir de ese momento, se estableció un contacto por correo electrónico o teléfono para programar la recopilación de datos. Es importante destacar que, antes del trabajo de campo, se realizó el reclutamiento y la formación del equipo, y la prueba piloto se realizó en febrero de 2023. La recopilación de datos tuvo lugar de marzo a diciembre de 2023, utilizando tabletas, donde el adolescente respondió por sí mismo al cuestionario estructurado en el sistema REDCap (*Research Electronic Data Capture*).

### Variables del estudio

El resultado en el presente estudio fue la violencia sexual en el noviazgo, que se dicotomizó (sí/no). La variable - violencia sexual en el noviazgo- se consideraba afirmativa si el adolescente había experimentado al menos una de las siguientes situaciones: “la pareja te acarició sin tu consentimiento ni voluntad, te obligó a tener relaciones sexuales cuando no querías, impidió el uso de preservativos, indujo el aborto o cualquier otro problema que vulnerara tus derechos sexuales y reproductivos”.

En cuanto a las variables independientes, se analizaron las variables sociodemográficas: sexo (mujer/hombre); grupo de edad (≤15 años/≥16 años); cisgénero (sí/no); heterosexual (sí/no); raza/color (blanco/negro/marrón); La clasificación económica medida por el Criterio de Clasificación Económica de la Asociación Brasileña de Empresas de Investigación (ABEP), que se clasifica como A/B/C/D/E; tiene un trabajo/trabajo (no/sí); y religión (ninguna/católica/evangélica/otras). También se analizaron las siguientes variables conductuales: relaciones sexuales en algún momento de la vida (no/sí); Uso actual de cigarrillos (no/sí); uso actual de drogas (no/sí) y uso actual de alcohol (no/sí), así como las variables relacionadas con las experiencias de violencia: sufrir *cyberbullying* en algún momento de la vida (no/sí); *cyberbullying* al menos una vez en su vida (no/sí); sufrió *bullying* en algún momento de tu vida (no/sí); practicó *bullying* en algún momento de su vida (no/sí).

### Tratamiento y análisis de datos

Para los análisis estadísticos descriptivos, se calcularon frecuencias absolutas y relativas. En el análisis bivariado, se utilizó la prueba de chi-cuadrado de Pearson (χ2) según supuestos, con sus respectivos intervalos de confianza del 95%. Para un análisis multivariante bruto ajustado por la razón de probabilidades a través de la regresión logística para evaluar las asociaciones entre variables independientes y violencia sexual en las citas, inicialmente se realizó análisis descriptivo y bivariante con el cálculo de la razón de probabilidades (OR) bruta. Las variables con p ≤0,20 se incluyeron en el modelo multivariante, ajustadas por factores de confusión, y el valor p ≤ 0,05 permaneció en el modelo (Figura 1).

**Figura 1:** Modelo de análisis en niveles: diagrama de flujo

*ABEP = *Associação Brasileira de Empresas de Pesquisa*

Los resultados se presentaron como razones de probabilidad ajustadas (ORa) e intervalos de confianza del 95% (IC 95%). Los análisis se realizaron utilizando el *software* Stata 17.0, garantizando la robustez en la identificación de factores asociados.

### Consideraciones éticas

Preservando cuestiones éticas, el estudio fue enviado al Comité de Ética en Investigación de la Universidade Federal do Espírito Santo y se llevó a cabo tras la aprobación y registro bajo el dictamen 5.900.370.

## Resultados

La Tabla 1 muestra la prevalencia de la violencia sexual en el noviazgo entre adolescentes de la Educación Secundaria. Se observa que, de los 1.238 adolescentes participantes en la encuesta, el 7,8% (IC 95%: 6,4-9,4) declaró haber sufrido ya violencia sexual en el noviazgo. Al analizar los resultados por género, el 9,3% (IC 95%: 7,4-11,5) de las adolescentes reportaron experimentar este tipo de violencia, mientras que entre los adolescentes del sexo masculino la prevalencia fue del 5,1% (IC 95%: 3,4-7,6).

**Tabla 1:** Prevalencia de la violencia sexual en el noviazgo entre adolescentes de la Educación Secundaria en la Región Metropolitana de Vitória, ES, Brasil, 2023

	Muestra general			Feminino			Masculino		
	N*	%^†^	IC95%^‡^	N*	%^†^	IC95%^‡^	N*	%^†^	IC95%^‡^
Violencia sexual en el noviazgo (n=1.238)									
Sí	96	7,8	6,4-9,4	73	9,3	7,4-11,5	23	5,1	3,4-7,6
No	1142	92,3	90,6-93,6	715	90,7	88,5-92,6	427	94,9	92,4-96,6

*N = Frecuencia absoluta; ^†^% = Frecuencia relativa; ^‡^IC95% = Intervalo de Confianza de 95%

La Tabla 2 muestra que existe una relación entre la violencia sexual en las citas y las variables: sexo, heterosexualidad, religión, relaciones sexuales al menos una vez en la vida, uso actual de drogas y uso actual de alcohol, así como entre quienes han sufrido y practicado *cyberbullying* y sufrieron *bullying* (p<0.05).

**Tabla 2:** Distribución de la violencia sexual entre adolescentes de la Educación Secundaria de la Región Metropolitana de Vitória, ES, Brasil, 2023

Variables	Sí*	IC95%^†^	Valor p^‡^
Sociodemográficas			
**Sexo (n=1.238)**			0,009
Femenino	73 (9,3)	7,4-11,5	
Masculino	23 (5,1)	3,4-7,6	
**Grupo de edad (n=1.238)**			0,277
≤15 años	14 (6,0)	3,6-9,9	
≥16 años	82 (8,2)	6,6-10,1	
**Cisgénero (n=1.237)**			0,170
Sí	84 (7,4)	6,0-9,0	
No	11 (11,2)	6,3-19,2	
**Heterosexual (n=1.238)**			0,001
Sí	60 (6,4)	5,0-8,1	
No	36 (12,2)	8,9-16,5	
**Raza/color (n=1.195)**			0,544
Blanca (o)	34 (7,2)	5,2-10,0	
Negra (o)	21 (9,6)	6,4-14,3	
Parda (o)	39 (7,7)	5,7-10,4	
**ABEP^§^ (n=1.237)**			0,067
A	9 (4,1)	2,1-7,7	
B	45 (8,1)	6,1-10,7	
C/D/E	42 (9,1)	6,8-12,1	
**Tiene trabajo/empleo (n=1.238)**			0,442
No	62 (7,4)	5,8-9,3	
Sí	34 (8,6)	6,2-11,8	
**Religión (n=1.238)**			0,022
Ninguna	22 (7,1)	4,7-10,6	
Católica	16 (5,3)	3,3-8,5	
Evangélica	42 (8,1)	6,0-10,8	
Otras	16 (14,4)	9,0-22,3	
**Comportamentales**			
**Relación sexual en algún momento de la vida (n=1.238)**			0,001
No	22 (4,6)	3,0-6,9	
Sí	74 (9,7)	7,8-12,1	
**Uso actual de cigarrillos (n=402)**			0,220
No	35 (10,4)	7,5-14,1	
Sí	10 (15,6)	8,6-26,7	
**Uso actual de drogas (n=1.238)**			<0,001
No	71 (6,5)	5,2-8,2	
Sí	25 (16,7)	11,5-23,5	
**Uso actual de alcohol (n=1.238)**			0,002
No	46 (5,9)	4,5-7,8	
Sí	50 (10,8)	8,3-14,0	
**Experiencias de Violencias**			
**Sufrió *cyberbullying* alguna vez en la vida (n=1.238)**			0,001
No	80 (7,0)	5,7-8,7	
Sí	16 (16,0)	10,0-24,5	
**Practicó *cyberbullying* alguna vez en la vida (n=1.238)**			0,018
No	86 (7,3)	6,0-9,0	
Sí	10 (15,4)	8,5-26,3	
**Sufrió *bullying* alguna vez en la vida (n=1.171)**			<0,001
No	22 (4,0)	2,6-5,9	
Sí	68 (11,1)	8,8-13,8	
**Practicó *bullying* alguna vez en la vida (n=1.238)**			0,165
No	77 (7,3)	5,9-9,1	
Sí	19 (10,3)	6,6-15,5	

*Sí = Frecuencia absoluta y frecuencia relativa; ^†^IC95% = Intervalo de Confianza de 95%; ^‡^Valor p = Nivel de significación; ^§^ABEP = *Associação Brasileira de Empresas de Pesquisa*

La Tabla 3 muestra que los adolescentes no heterosexuales presentaron aproximadamente el doble de probabilidades de sufrir violencia sexual en el noviazgo (ORa: 2,05; IC 95%: 1,32-3,16). En el mismo sentido, quienes ya habían tenido relaciones sexuales al menos una vez en su vida tenían aproximadamente el doble de probabilidades de ser abusados sexualmente (ORa: 1,91; IC 95%: 1,15-3,16). Los adolescentes que reportaron uso de drogas tenían 2,19 veces más probabilidades de ser abusados sexualmente por su pareja (IC 95%: 1,31-3,66). Otro resultado encontrado fue en relación con quienes sufrieron acoso escolar (ORa: 2,72; IC 95%: 1,63-4,52) y ciberacoso (ORa: 1,97; IC 95%: 1,07-3,64), lo que aumentó significativamente las probabilidades de sufrir violencia sexual en el noviazgo.

**Tabla 3:** Análisis bruto y ajustado de la violencia en el noviazgo sexual entre adolescentes de la Educación Secundaria en la Región Metropolitana de Vitória, ES, Brasil, 2023

Variables	OR*	IC95%^†^	Valor p^‡^	ORa^§^	IC95%^†^	Valor p^‡^
Sociodemográficas						
**Sexo (n=1.238)**			0,010			0,093
Femenino	1,90	1,17-3,07		1,55	0,93-2,58	
Masculino	1			1		
**Grupo de edad (n=1.238)**			0,279			-
≤15 años	1			-		
≥16 años	1,38	0,77-2,48		-	-	
**Cisgénero (n=1.237)**			0,173			0,778
Sí	1			1		
No	1,59	0,82-3,09		1,11	0,54-2,26	
**Heterosexual (n=1.238)**			0,001			0,001
Sí	1			1,0		
No	2,05	1,32-3,16		2,05	1,32-3,16	
**Raza/color (n=1.195)**			0,545			-
Blanca (o)	1			-	-	
Negra (o)	1,37	0,77-2,42		-	-	
Parda (o)	1,07	0,66-1,72		-	-	
**ABEP^||^ (n=1.237)**			0,076			0,063
A	1			1		
B	2,06	0,99-4,30		2,18	1,04-4,55	
C/D/E	2,35	1,12-4,92		2,42	1,15-5,08	
**Tiene un trabajo/empleo (n=1.238)**			0,443			-
No	1			-	-	
Sí	1,19	0,77-1,84		-	-	
**Religión (n=1.238)**			0,027			0,075
Ninguna	1			1		
Católica	0,74	0,38-1,43		0,96	0,48-1,90	
Evangélica	1,15	0,67-1,97		1,44	0,82-2,54	
Otras	2,20	1,11-4,36		2,22	1,10-4,46	
**Comportamentales**						
**Relación sexual alguna vez en la vida (n=1.238)**			0,001			0,012
No	1			1		
Sí	2,24	1,37-3,65		1,91	1,15-3,16	
**Uso actual de cigarrillos (n=402)**			0,224			-
No	1			-	-	
Sí	1,60	0,75-3,43		-	-	
**Uso actual de drogas (n=1.238)**			<0,001			0,003
No	1			1		
Sí	2,86	1,75-4,69		2,19	1,31-3,66	
**Uso actual de alcohol (n=1.238)**			0,002			0,405
No	1			1		
Sí	1,93	1,27-2,93		1,22	0,76-1,96	
**Experiencias de Violencias**						
**Sufrió *cyberbullying* alguna vez en la vida (n=1.238)**			0,002			0,029
No	1			1		
Sí	2,52	1,41-4,50		1,97	1,07-3,64	
**Practicó *cyberbullying* alguna vez en la vida (n=1.238)**			0,021			0,219
No	1			1		
Sí	2,30	1,13-4,67		1,63	0,75-3,57	
**Practicó *bullying* alguna vez en la vida (n=1.238)**			<0,001			<0,001
No	1			1		
Sí	3,03	1,85-4,97		2,72	1,63-4,52	
**Practicó *bullying* alguna vez en la vida (n=1.238)**			0,167			0,568
No	1			1		
Sí	1,45	0,86-2,46		1,19	0,66-2,15	

*OR = *Odds Ratio* bruto; ^†^IC95% = Intervalo de Confianza de 95%; ^‡^Valor p = Nivel de significación; ^§^ORa = *Odds Ratio* Ajustado; ^||^ABEP = *Associação Brasileira de Empresas de Pesquisa*

## Discusión

La prevalencia general de violencia sexual en el noviazgo entre adolescentes en la educación secundaria, encontrada en este estudio, fue del 7,8% (IC 95%: 6,4-9,4), lo que indica que una parte considerable de los adolescentes experimenta violencia sexual en sus relaciones afectivas, y representa una realidad preocupante, considerando los impactos de la violencia sexual en esta etapa del desarrollo. En Estados Unidos, una encuesta realizada por los *Centers for Disease Control and Prevention* (CDC) identificó que aproximadamente el 7,5% de los adolescentes (IC 95%: 6,4-8,7) habían sufrido violencia sexual en el noviazgo^10^. En Brasil, los datos de la *Pesquisa Nacional de Saúde do Escolar* (PeNSE) indican que aproximadamente el 4% de los adolescentes reportaron haber sido forzados a mantener relaciones sexuales, siendo su novio o exnovio el principal agresor^11^.

Para las niñas, la prevalencia de violencia sexual en el noviazgo fue del 9,3% (IC 95%: 7,4-11,5) y del 5,1% (IC 95%: 3,4-7,6) en los niños. Un estudio sobre violencia sexual en escolares en Brasil, con datos de la encuesta nacional de salud escolar de 2019, mostró una prevalencia de violencia sexual en el noviazgo del 8,8% entre mujeres (IC 95%: 8,4-9,3) y en hombres, 3,7% (IC 95%: 3,3 - 4,0)^12^. En Estados Unidos, los CDC identificaron una disparidad significativa de género en la violencia sexual en el noviazgo, 11,3% de niñas frente a 3,5% de chicos, lo que indica que las adolescentes tienen un riesgo tres veces mayor^13^. Es un hecho que las niñas son más vulnerables a este tipo de violencia, debido a factores como la desigualdad de género, la socialización basada en la sumisión femenina y las normas culturales que toleran conductas abusivas por parte de las parejas afectivas^14^.

El presente estudio mostró que los adolescentes no heterosexuales tenían aproximadamente el doble de probabilidades de sufrir violencia sexual en el noviazgo (ORa: 2,05; IC 95%: 1,32-3,16). En consonancia, los datos del *Youth Risk Behavior Survey* de 2021, realizada en Estados Unidos, muestran que los adolescentes que se identifican como LGB reportaron, en mayor proporción que sus compañeros heterosexuales, experiencias de violencia sexual en el noviazgo^15^. Específicamente, el 17% de los adolescentes LGBTQ+ afirmaron que ya habían sido obligados a mantener relaciones sexuales, en comparación con el 6% de los adolescentes heterosexuales^10^. Esta evidencia revela que pertenecer a minorías sexuales representa un factor de riesgo adicional para la violencia en el noviazgo, especialmente la violencia sexual. Los adolescentes LGBTQIA+ están más expuestos a diversas formas de violencia y vulnerabilidad debido a la discriminación estructural, el estigma social y el rechazo familiar, lo que contribuye a la invisibilidad y la subdenuncia de estos casos^16^.

Estas violencias, a menudo naturalizadas o descuidadas, ocurren no solo en el ámbito relacional, sino también en instituciones, como escuelas y servicios de salud, que no siempre están preparados para acoger y proteger a estas poblaciones^17^. Además, la falta de reconocimiento de la identidad de género y la invalidación de la orientación sexual por parte de las familias intensifican el sufrimiento psicológico, incrementando el riesgo de depresión, ansiedad, consumo de sustancias e ideación suicida^18^.

Además, los resultados del presente estudio indican que los adolescentes que ya han comenzado su vida sexual tienen más probabilidades de sufrir violencia sexual en el contexto del noviazgo (ORa: 1,91; IC 95%: 1,15-3,16). Este hallazgo es coherente con la literatura reciente, que asocia la iniciación sexual temprana o no planificada con una serie de vulnerabilidades sociales y conductuales que pueden favorecer situaciones de abuso. Según el estudio, los adolescentes que comenzaron su vida sexual a los 13 años o menos tenían una mayor prevalencia de conductas sexuales de riesgo, incluyendo menor uso de preservativos y mayor exposición a la violencia sexual^(19^).

El consumo actual de drogas también se asoció con un aumento de 2,19 veces en la probabilidad de ser abusado sexualmente por parte de una pareja (IC 95%: 1,31-3,66). En una revisión bibliográfica, destacan que la violencia en el noviazgo entre adolescentes representa un problema relevante para la salud pública, influenciada por factores como el consumo de sustancias psicoactivas, que pueden contribuir al aumento de conductas agresivas en las relaciones en este grupo de edad^20^. Además, un estudio realizado en Nicaragua con 1.799 adolescentes reveló que la prevalencia de la violencia en las citas estaba asociada con el uso de sustancias psicoactivas como el alcohol, los cigarrillos tradicionales, la marihuana, los cigarrillos electrónicos y conductas externas como la agresividad, la desobediencia, los comportamientos antisociales y otros comportamientos disruptivos. Los resultados indican que los adolescentes que consumen sustancias psicoactivas tienen más probabilidades de involucrarse en relaciones abusivas^20^.

Se observó que la experiencia del *bullying* está asociada con una mayor vulnerabilidad de los adolescentes ante experiencias de violencia y relaciones interpersonales marcadas por la coerción y el abuso, lo que indica la posibilidad de transferir la victimización entre diferentes contextos sociales. La evidencia poblacional muestra que los adolescentes expuestos al *bullying* tienen más probabilidades de participar en conductas de riesgo y relaciones interpersonales poco saludables, aumentando su susceptibilidad a experiencias abusivas a lo largo del desarrollo^21^ ^-^ ^22^. De manera similar, un estudio identificó la relación entre el acoso escolar y la violencia entre parejas adolescentes, destacando que quienes han experimentado situaciones de agresión o humillación en el entorno escolar tienen más probabilidades de tolerar conductas abusivas en sus relaciones^19^.

Las limitaciones de la investigación actual son la naturaleza transversal de la investigación, ya que captura un momento único en el tiempo, impidiendo análisis causales robustos, y también es susceptible a sesgos de recuerdo en los instrumentos de recopilación. Sin embargo, la investigación ofrece contribuciones sustanciales al ser pionera en el mapeo de los patrones de violencia en el noviazgo entre jóvenes en una importante metrópoli brasileña, proporcionando datos representativos para esta población específica.

## Conclusión

La prevalencia del 7,8% de la violencia sexual en el noviazgo identificada entre los adolescentes participantes en este estudio resalta la relevancia y urgencia de enfrentarse a este tipo específico de violencia en las relaciones afectivas juveniles. Estos datos refuerzan que la victimización sexual ya está presente en las primeras experiencias amorosas.

El presente estudio demostró que la violencia sexual en el noviazgo entre adolescentes está significativamente asociada con factores como el consumo actual de sustancias psicoactivas, adolescentes no heterosexuales, quienes han tenido relaciones sexuales al menos una vez en sus vidas y quienes han sufrido *bullying* y *cyberbullying*. Estos hallazgos indican que la victimización sexual en el contexto de las relaciones afectivas entre adolescentes ocurre de forma concurrente con otras experiencias de exposición a la violencia y conductas de riesgo, lo que pone de manifiesto la importancia de estrategias de prevención que tengan en cuenta la complejidad de estas asociaciones y la coexistencia de diferentes formas de vulnerabilidad en la adolescencia.

Tales evidencias son especialmente relevantes para los profesionales de la salud, la educación y la asistencia social, así como para los responsables de políticas públicas, ya que señalan grupos y contextos que exigen mayor atención en acciones de prevención y protección dirigidas a las relaciones afectivas juveniles. El reconocimiento de estos factores asociados puede contribuir a la dirección de estrategias intersectoriales que promuevan relaciones más seguras, igualitarias y respetuosas entre los adolescentes.

Por ello, los resultados de este estudio reiteran la importancia de desarrollar e implementar políticas y programas para prevenir la violencia sexual en el noviazgo, con énfasis en reducir los factores asociados identificados y promover relaciones afectivas saludables entre los adolescentes.

## Data Availability

Todos los datos generados o analizados durante este estudio están incluidos en este artículo publicado.
